# Oral Cancer Theranostic Application of FeAu Bimetallic Nanoparticles Conjugated with MMP-1 Antibody

**DOI:** 10.3390/nano12010061

**Published:** 2021-12-27

**Authors:** Meng-Tsan Tsai, Ying-Sui Sun, Murugan Keerthi, Asit Kumar Panda, Udesh Dhawan, Yung-Hsiang Chang, Chih-Fang Lai, Michael Hsiao, Huey-Yuan Wang, Ren-Jei Chung

**Affiliations:** 1 Department of Electrical Engineering, Chang Gung University, 259, Wenhua 1st Rd., Taoyuan City 33302, Taiwan; mttsai@mail.cgu.edu.tw; 2 Department of Neurosurgery, Chang Gung Memorial Hospital, Linkou Branch, 5, Fuxing St., Guishan Dist., Taoyuan City 33305, Taiwan; 3 School of Dental Technology, College of Oral Medicine, Taipei Medical University, 250, Wu-Hsing St., Taipei 11031, Taiwan; yingsuisun@tmu.edu.tw; 4 Department of Chemical Engineering and Biotechnology, National Taipei University of Technology (Taipei Tech), 1, Sec. 3, Zhongxiao E. Rd., Taipei 10608, Taiwan; keerthimurugan1992@gmail.com (M.K.); asitpanda6@gmail.com (A.K.P.); a67621p@gmail.com (Y.-H.C.); 5 Centre for the Cellular Microenvironment, University of Glasgow, Glasgow G12 8QQ, UK; 6 DFON Biomedical Technology Inc., 1, Sec. 3, Zhongxiao E. Rd., Taipei 10608, Taiwan; Joshua@dermafountain.com; 7 Genomics Research Center, Academia Sinica, 128, Sec. 2, Academia Rd., Nankang, Taipei 115, Taiwan; mhsiao@gate.sinica.edu.tw; 8 Department of Biochemistry, College of Medicine, Kaohsiung Medical University, No. 100, Shih-Chuan 1st Rd., Sanmin Dist., Kaohsiung City 80708, Taiwan; 9 Department of Stomatology, MacKay Memorial Hospital, 92, Sec. 2, Zhongshan N. Rd., Taipei 10449, Taiwan

**Keywords:** oral squamous cell carcinoma, cancer theranostic, iron–gold bimetallic nanoparticles, matrix metalloproteinase-1, magnetic hyperthermia

## Abstract

Metastatic oral squamous cell carcinoma (SCC) displays a poor disease prognosis with a 5-year survival rate of 39%. Chemotherapy has emerged as the mainstream treatment against small clusters of cancer cells but poses more risks than benefits for metastatic cells due to the non-specificity and cytotoxicity. To overcome these obstacles, we conjugated antibodies specific for matrix metalloproteinase-1 (MMP-1), a prognostic biomarker of SCC, to iron–gold bimetallic nanoparticles (FeAu NPs) and explored the capability of this complex to target and limit SSC cell growth via magnetic field-induced hyperthermia. Our results showed that 4.32 ± 0.79 nm sized FeAu NPs were superparamagnetic in nature with a saturation magnetization (Ms) of 5.8 emu/g and elevated the media temperature to 45 °C, confirming the prospect to deliver hyperthermia. Furthermore, conjugation with MMP-1 antibodies resulted in a 3.07-fold higher uptake in HSC-3 (human tongue squamous cell carcinoma) cells as compared to L929 (fibroblast) cells, which translated to a 5-fold decrease in cell viability, confirming SCC targeting. Finally, upon magnetic stimulation, MMP-1-FeAu NPs conjugate triggered 89% HSC-3 cellular death, confirming the efficacy of antibody-conjugated nanoparticles in limiting SCC growth. The synergistic effect of biomarker-specific antibodies and magnetic nanoparticle-induced hyperthermia may open new doors towards SCC targeting for improved disease prognosis.

## 1. Introduction

Oral cancer is a common term for malignant tumors that occur in the oral cavity; about 90% of them are squamous cell carcinoma [1]. It is reported that oral cancer occurs in different parts of the mouth, such as the buccal mucosa (32%), tongue (22%), lower lip (11%), palate (11%), the vestibule (8%), alveolus (5%), floor of the mouth (5%), and gingiva (3%) [2,3]. The major risk factors for oral cancer include an unhealthy lifestyle, immune system suppression, genetic factors, chronic trauma, and human papillomavirus [4]. The lifestyle factors that are associated with the risk of developing oral cancer are smoking, betel nut or tobacco chewing, and poor oral hygiene [5,6]. According to the statistics of the Ministry of Health and Welfare and the National Cancer Agency, every year about 7000 new cases are reported and about 3000 people die because of oral cancer, among which 90% are male [3,7]. The current treatments for oral cancer include surgery, radiation therapy, and taking anticancer drugs [8,9]. Recently, magnetic hyperthermia therapy (MHT) has gained increasing attention owing to its non-invasiveness, high effectiveness, excellent tissue penetration, and cost effectiveness [10,11,12]. This treatment modality makes use of small magnetic nanoparticles (MNPs), that not only display surface effect and quantum size effect but also have superparamagnetic properties [13,14]. MNPs have a larger surface area which facilitates their modification by antibodies to enhance tumor specificity while reducing toxicity towards the surrounding tissues. The metabolism of tumor cells is comparatively faster than that of normal cells making the former more reactive to any change in pH and surrounding temperature [15,16]. It has been reported that cancer cells are destroyed at temperatures in the range of 41–45 °C while healthy cells can withstand these temperatures. Upon excitation with an alternating magnetic field (AMF), MNPs are capable of transforming electromagnetic radiation (EMR) to heat, and the heat generated can be utilized to destroy cancer cells [17,18]. The inner region of the MNPs has an internal resistance, and the current flow through the tissue causes energy loss, thereby generating heat to target cancer cells. Hence, MNPs can be utilized as carriers of hyperthermic treatment for cancers [19,20].

The bimetallic nanoparticles are more efficient than the corresponding single metal component nanoparticles due to their chemical and physical diverse properties. FeAu NPs have multifunctional characteristics, including optical and magnetic properties [21]. Furthermore, the presence of gold allows for the ease of functionalization with biomolecules such as nerve growth factor, as shown by Yuan et al. [22]. Other attractive properties of using FeAu bimetallic nanoparticles include their ability to degrade into smaller particles in a physiological environment which facilitates their clearance by the body [23]. Finally, as shown by Song et al., the presence of Au allows functionalization with polymeric materials for the formation of amphiphilic nanomaterials [24]. In this study, FeAu bimetallic NPs were prepared by the thermal pyrolysis method. We previously found that FeAu NPs are capable of generating 1.33 × 10^6^ J of heat energy per mole in the presence of high-frequency induction waves (HFIW) [25,26]. Moreover, FeAu bimetallic NPs prepared by this method have excellent biocompatibility, superior optical properties, superparamagnetic effects, and suitable monodispersity in water, and can be easily functionalized with sulfur-containing biomolecules [21,27]. FeAu bimetallic NPs are an excellent choice for magnetic guidance, and magnetothermal therapy [28]. Thus, the matrix metalloproteinase-1 (MMP-1) antibody was modified on the surface of FeAu bimetallic NPs to act as a target for MMP-1 targeting high concentrations in and out of oral cancer cells. The matrix metalloproteinases (MMPs), zinc-dependent endopeptidases are primarily involved in remodeling of extracellular matrix (ECM) [29,30]. MMP-1 is also known as interstitial collagenase and fibroblast collagenase, which is responsible for the digestion of type I, II, and III natural fibrillar collagen in the extracellular environment [31,32]. According to previous research, the high expression of MMP-1 in cancer is relevant, as it is particularly helpful during the invasion and metastasis of tumor cells [33,34,35,36]. Thus, we present a novel approach to conjugate MMP-1 antibodies for targeting oral carcinoma cells followed by their cellular death via magnetothermal treatment.

## 2. Materials and Methods

### 2.1. Material and Characterization

Didecyldimethyl ammonium bromide (DDAB), toluene 99%, C_6_H_5_CH_3,_ ferrous sulfate heptahydrate (FeSO_4_·7H₂O), sodium borohydride (NaBH_4_) 99.9%, tetrachloroauric (III) acid trihydrate (HAuCl_4_·4H_2_O), 3-mercapto-1-propanesulfonic Acid 90%, absolute ethanol 99.9%, L-cysteine, cysteamine, and all other chemicals of analytical grade were purchased from Sigma or Merck. EDC, Sulfo-NHS, and MES Buffer were obtained from R&D Systems. MMP-1 antibody was acquired from R&D Systems. Transmission electron microscopy (TEM, JEM2100F/JEOL/, Tokyo, Japan) was used for the morphological analysis of FeAu NPs and MMP-1 antibody-conjugated FeAu NPs. The particle size and surface potential of nanoparticle-peptide conjugation were studied using Image J and Zetasizer (JEM2100F/JEOL/, Tokyo, Japan), respectively. X-ray diffraction (XRD) spectroscopy (X’Pert^3^ powder/PANalytical/, Almelo, The Netherlands) was used to observe the crystalline structure of the FeAu NPs while the composition was confirmed using energy dispersive X-ray spectroscopy (EDS). FTIR (FT720/Horiba/, Kyoto, Japan) and raman spectroscopy (DongWoo) studies were used to confirm the conjugation of MMP-1 antibodies with FeAu NPs. Magnetic properties were analyzed using a superconducting quantum interference device (SQUID;MPMS3/Quantum Design/, San Diego, CA, USA).

### 2.2. Synthesis of Iron-Gold (FeAu) Bimetallic NPs

FeAu bimetallic NPs were prepared via thermal pyrolysis method. Ferrous sulfate heptahydrate (FeSO_4_·7H₂O) and tetrachloroauric (III) acid trihydrate (HAuCl_4_·4H_2_O) were used as the Fe and Au precursors, respectively. Toluene was used as a solvent, didecyldimethyl ammonium bromide (DDAB), and sodium borohydrate (NaBH_4_) were used as protecting and reducing agents, respectively. Initially, 20 mL of toluene containing 0.16 mM of dodecyl dimethyl was taken in a three-necked flask. Meanwhile, N_2_ gas was passed into the solvent to expel excess oxygen, and the temperature was maintained at 110 °C using a thermocouple thermometer under constant magnetic stirring for 15 min. After that, 0.08 mM of FeSO_4_·7H₂O was dissolved in 1 mL of DI water and injected into the above solvent. The color of the solution changed and turbidity appeared. After 2 min, 1 mL of 0.015 M NaBH_4_ was added to the solution to reduce Fe, which was confirmed by observing the formation of a black suspension. Stirring was continued for another 20 min. Later, 21.2 mM of 3-fluorenyl-1-propane sodium sulfate was dissolved in 0.013 M of HAuCl_4_·4H_2_O, and the color of the solution was observed to change from yellow to colorless. This colorless Au solution was injected into a three-necked bottle and the color of the solution turned red. A total of 1.5 mL of 0.015 M NaBH_4_ was added to the mixture to reduce HAuCl_4_·4H_2_O to Au. At this time of injection, the solution displayed a purple color, slowly turning into a light reddish color. The solution was then stirred for 30 min. Next, 1 mL of 0.515 M NaBH_4_ was again injected into the same solution, and the stirring was stopped. The temperature of the solution was maintained at 84 °C for 3 h. Finally, the synthesized product was separated via centrifugation at 9000 rpm for 15 min and washed with ethanol. After centrifugation, the FeAu MNPs were collected using 4000 G magnet and dried for 4 h using a vacuum pump.

### 2.3. Surface Modification and Synthesis of MMP-1 Antibody Conjugated FeAu NPs

For surface modification, L-cysteine (L-cys) and FeAu NPs were taken in 1:10 weight percent, dispersed in a weakly alkaline solution containing 30 mL of water and 1 mL of 1 M NaOH. The above solution was stirred for 24 h at 25 °C to ensure that L-cys successfully modified the FeAu NPs through the formation of bonds between sulfur of L-cys and Au of FeAu NPs. For conjugation of MMP-1 antibodies to FeAu NPs, 10 mg of FeAu NPs and 1 mg of MMP-1 antibodies were added into 20 mL PBS (pH 7.4) and then stirred for 24 h under an inert atmosphere. The mixture was ultrasonicated until a uniform dispersion was formed. Subsequently, the dispersion was centrifuged at 4000 rpm for 20 min and repeated several times to remove unbound MMP-1 antibodies. Finally, MMP-1 antibody-conjugated FeAu NPs were magnetically collected using a 4000 G magnet and dispersed in PBS. The antibody-nanoparticle conjugates were dried using a vacuum pump.

### 2.4. Magnetic Stimulation-Induced Concentration-Dependent Temperature Elevation

The ability of NPs to generate heat with various concentrations was studied using an alternating magnetic field (AMF, 700–1100 KHz) for 10 min. For this, NPs were prepared with different concentrations (0.5, 1, 2.5, 5 mg/mL) in water.

### 2.5. Cell Culture

Mouse fibroblasts (L929) and human oral squamous cell carcinoma (HSC-3) were used for in vitro experiments in this study. L929 and HSC-3 cells were cultured in Dulbecco’s modified eagle medium (DMEM) and modified eagle medium (MEM), respectively. Cells were cultured in T75 flasks in an incubator maintained at 37 °C, with a 5% CO_2_ atmosphere. Cells were passaged every 2 to 3 days.

### 2.6. In Vitro Cytotoxicity Analysis

In vitro cytotoxicity was evaluated using the MTT (3-(4,5-dimethylthiazol-2-yl)-2,5-diphenyltetrazolium bromide) assay. A total of 5 mg/mL MTT reagent was prepared and stored at 4 °C in dark. First, the stock solution was diluted 10-fold using the culture medium. The cells were seeded at a density of 5 × 10^4^ cells/mL in 24-well plates for 24 h to evaluate the in vitro cytotoxicity of FeAu or MMP-1 antibody conjugated FeAu NPs. After 24 h of incubation, culture media was removed and cells were washed twice using pre-warmed PBS. 50 µL of MTT reagent in a 0.5 mL culture medium was added to cells and incubated for 4 h in a dark environment. After 4 h, MTT reagent was removed from the culture medium and 1 mL of DMSO was added to dissolve the purple crystals. After shaking using a rotary shaker at 100 rpm for 10 min, 200 uL was taken from each experimental group and transferred to 96-well plates. Absorbance was measured at 570 nm. A total of 0.1 g of Teflon was taken as a negative control, 0.1 g latex was taken as a positive control, and DMEM medium was used as a blank. Cell viability was calculated using the following formula:

(1) $$\mathrm{Cell}\;\mathrm{viability}=\frac{Absorbance\;\left(Sample\right)-Absorbance\;\left(Blank\right)}{Absorbance\;\left(Control\right)-Absorbance\;\left(Blank\right)}\times 100$$

### 2.7. The Evaluation of Cell-Specific MMP-1 Antibody-Conjugated FeAu NPs Ingestion

MMP-1 antibody-conjugated FeAu NPs were used in this experiment. The cells were seeded at a density ot 3 × 10^5^ cells/mL into a 35 mm petri dish with 1 mL of culture solution and cultured in an incubator for 24 h. The next day, the purified FeAu NPs and MMP-1 antibody-conjugated FeAu NPs were irradiated with UV light for 30 min. MEM was configured to a concentration of 100 µg/mL; 1 mL was added into the petri dish and incubated for 0.5 and 2 h. After washing three times with PBS, 0.5 mL of trypsin-EDTA was added to each well. After the cells were purified via centrifugation, 1 mL of concentrated nitric acid was added to the cells. Then DI water was added to the media for a 10-fold dilution, and the iron concentration was measured using inductively coupled plasma atomic emission spectrometry (ICP-AES).

### 2.8. The Effect of Magnetic Field-Induced Hyperthermia on HSC-3

To understand the therapeutic effect of magnetic field-induced hyperthermia on oral cancer cells, HSC-3 cells were cultured in a 35 mm culture dish with a cell density of 3 × 10^5^ cells/mL for 24 h. After 24 h, the culture solution was removed and discarded. FeAu NPs and MMP-1-FeAu NPs were sterilized using UV light for 30 min and then diluted in culture medium at a concentration of 100 μg/mL. A total of 1 mL of this concentration was added to each culture dish. The cells were incubated with FeAu NPs and MMP-1-FeAu NPs for 4 h. After 4 h, samples were subjected to alternating magnetic field stimulation (AMF) for 0, 5, 10, and 15 min, and then placed in the incubator for 24 h. On the third day, MTT reagent was added in a darkened environment. After 4 h, the reagent was removed and 1 mL of DMSO was added to each well and placed on an orbital shake at 100 RPM for 10 min. Later, 200 µL of the solution from each dish was transferred to a 96-well plate. The absorbance was measured at 570 nm using an ELISA reader.

### 2.9. Analysis of Nanoparticle Ingestion Using Bio-TEM

Bio-TEM analysis was performed to estimate the intracellular localization of MMP-1 antibody-conjugated FeAu NPs or lone FeAu NPs. HSC-3 cells were seeded at a density of 3 × 10^6^ cells/mL in 2 wells of a multi-well culture plate. The cells were allowed to attach and grow for 24 h. The next day, the purified FeAu NPs and MMP-1 antibody-conjugated FeAu NPs were irradiated with UV light for 30 min. MEM was configured to a concentration of 100 µg/mL and added to each well containing HSC-3 cells for 24 h. The cells were kept in a fixative solution containing paraformaldehyde (2%) and glutaraldehyde (2.5%) in 0.1 M cacodylate. Subsequently, the samples were dipped in PBS buffer followed by dehydration and then vacuum drying. The intracellular location of nanoparticles was analyzed using TEM.

### 2.10. In Vivo Mouse Model for Evaluation of Anti-Cancer Treatment

A total of 15 4-week-old Balb/c Nude mice (weight = 18–22 g) were employed for this study. The animals were acclimatized for at least one week prior to the experiment. The experiments were carried out at MacKay Memorial Hospital with their guidelines to the care and use of animals. All the protocols for animal study and use were approved by the affiliated Institutional Animal Care and Use Committee (IACUC) under the affidavit no. MMH-A-S-108-16. The animals had ad libitum access to standard rat chow and water at all times. For surgical anesthesia, zoletil 50 (Virbac, Carros, France) was injected intraperitoneally at a dosage of 20 mg/Kg.

### 2.11. The Measurement of Tumor Growth in Nude Mice

To monitor the suppression of tumor growth in xenograft model mice, 4 week-old female immuno-deficient nude mice (Balb/c Nude) were used. HSC-3 cells (10^4^ cells/μL/mice) were injected subcutaneously into the hind limbs. The animals were acquired from BioLASCO Taiwan Co., Ltd. (Taipei, Taiwan), and kept in a specific pathogen-free (SPF) environment at the Laboratory Animal Unit.

### 2.12. The Growth of HSC-3 Cells Was Inhibited by Magnetic Hyperthermia Therapy

When the tumor volume reached around 40 mm^3^, the mice were randomly divided into three groups (n = 5 for each group) and intratumorally injected with FeAu NPs (0.2 mg/200 µL), antiMMP1-FeAu NPs (0.2 mg/200 µL) suspended in PBS, and PBS alone (control group). For the next 2 h, NPs were allowed to penetrate in tumor tissue via phagocytosis (the process by which a cell engulfs solid particles). Hereafter, to inhibit the tumor growth via the NPs injected, we used an alternating magnetic field (AMF, 700–1100 kHz) for 10 min. The average tumor volume was measured and recorded every 3 days for a period of 30 days after magnetic hyperthermia treatment. 

### 2.13. Statistics

All experiments were performed at least thrice using nanoparticles. Student’s two-tailed *t*-test was used to identify if the difference between experimental groups was statistically significant. The level of significance was set as 0.05 or 0.01. *p*-values ≤ 0.05 were denoted with * and *p*-values ≤ 0.01 were denoted with **.

## 3. Results and Discussions

### 3.1. The Characterization of FeAu Nanoparticles

**Figure 1:**
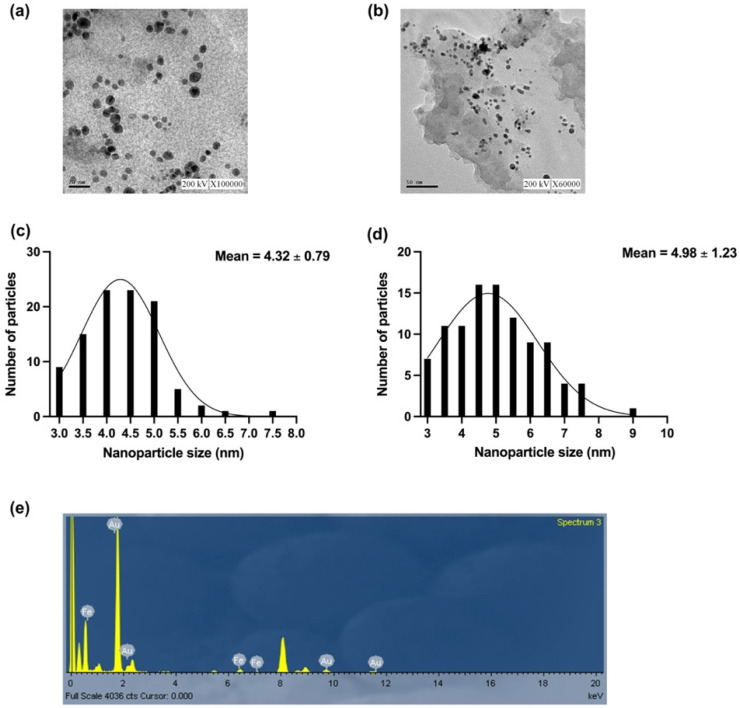
The characterization of FeAu and MMP-1 antibody-conjugated FeAu NPs. (**a**) TEM micrograph of FeAu NPs, (**b**) TEM micrograph of MMP-1 antibody-conjugated FeAu NPs, (**c**) size distribution of FeAu NPs, (**d**) size distribution of MMP-1 antibody-conjugated FeAu NPs, and (**e**) elemental composition of FeAu NPs analyzed using EDS analysis.

The morphological characteristics and particle size distribution of FeAu NPs and MMP-1 antibody-conjugated FeAu NPs were investigated by TEM and analyzed using Image J. The TEM image of the FeAu NPs showed a spherical structure (Figure 1a). In the case of MMP-1 antibody-conjugated FeAu NPs, similar spherical FeAu particles were observed without the agglomeration of NPs (Figure 1b). The average particle size of FeAu NPs was observed to be 4.32 ± 0.79 nm (Figure 1c). Additionally, the average particle size of MMP-1 antibody-conjugated FeAu NPs was 4.98 ± 1.23 nm, as shown in Figure 1d. TEM-EDS analysis was performed to further understand the compositional ratio of FeAu NPs. EDS analysis highlighted that (Figure 1e and Table 1), the weight percentage of Fe and Au obtained were 18.40 and 81.60 wt.%, respectively. Additionally, the ratio of theoretical weight percentage and the molar ratio of Fe and Au were found to be close to 1:1, as shown in Table 2.

**Figure 2:**
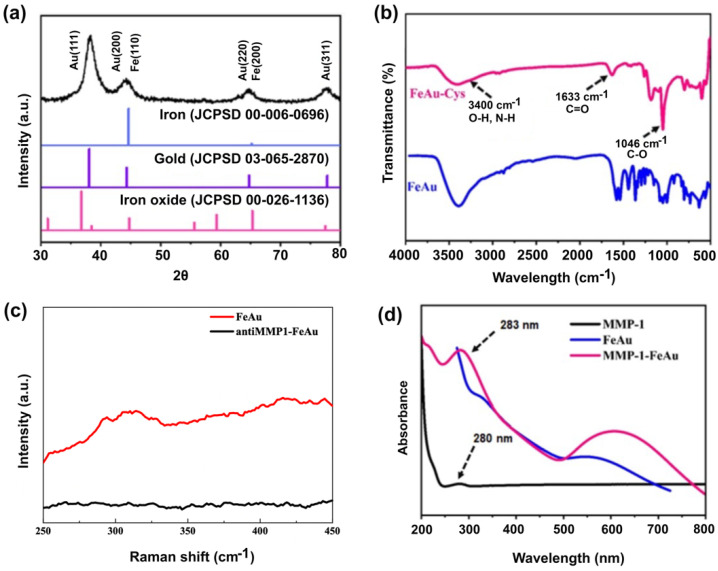
Confirmation of MMP-1 conjugation to FeAu nanoparticles. (**a**) X-ray diffraction pattern of FeAu NPs, (**b**) FTIR pattern of FeAu and FeAu-Cys, (**c**) Raman spectra before and after FeAu NPs modification, and (**d**) UV-Vis spectrum of FeAu NPs before and after MMP-1 antibody modification.

X-ray diffraction spectroscopy was used to analyze the crystalline phase of the FeAu bimetallic NPs (Figure 2a). The XRD pattern of FeAu bimetallic NPs shows the main peaks at 2Ɵ value at 44.67° and 65.02° corresponding to the crystal plane of (110) and (200), which is due to the body-centered cubic (BCC) structure of Fe (JCPDS 00-006-0696) and multiple peaks at 38.28°, 64° and 77.77° which correspond to the crystal planes of (111), (220) and (311), indicating the face-centered cubic (FCC) structure of Au (JCPDS 03-065-2870). Moreover, no additional peaks were observed for iron oxide, confirming the successful formation of FeAu bimetallic NPs.

### 3.2. The Confirmation of FeAu-Cys NPs Formation

FTIR was performed to confirm the conjugation of cysteine to FeAu NPs (Figure 2b). As can be seen in Figure 2b, upon comparing the spectra before and after modification, the band generated at 1000~1078 cm^−1^ is due to CO (stretch) and the absorption band at 1580~1710 cm^−1^ is due to the C=O signal, confirming the successful surface modification of FeAu NPs by cysteine. As can be seen from the Raman spectra (Figure 2c), FeAu-Cys NPs exhibit absorption peaks at 290 cm^−1^ to 330 cm^−1^, which is the absorption peak of Au–sulfur bonding. It occurs as a result of bond formation between the Au of NPs and the -SH group of cysteine, which further confirms the surface modification of FeAu NPs by cysteine. UV-Vis analysis was performed to investigate the conjugation of the MMP-1 antibody to FeAu NPs. From the UV-vis spectra (Figure 2d), it can be seen that the MMP-1 antibody has an absorption peak at a wavelength of 280 nm. Further, the UV-vis spectrum of MMP-1 antibody-conjugated FeAu NPs exhibits the characteristic absorption peak of MMP-1 antibody at a wavelength of 283 nm confirming successful conjugation of the MMP-1 antibody to FeAu NPs.

### 3.3. Confocal Analysis

**Figure 3:**
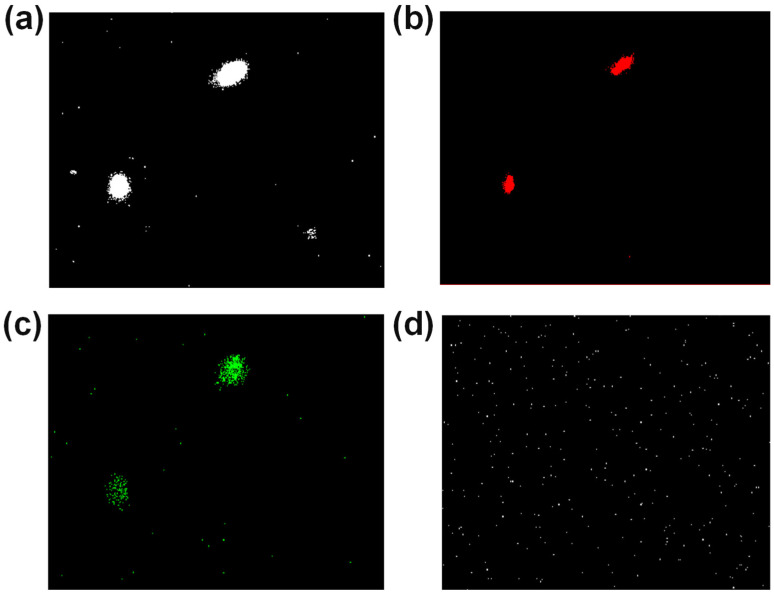
Confocal images of FeAu in different filters under 515 nm laser. (**a**) Null, (**b**) 600 ± 37 nm, (**c**) 530 ± 43 nm, (**d**) 440 ± 40 nm.

It is known through UV-Vis spectroscopy that FeAu NPs have an absorption peak at 600 nm. After exciting FeAu NPs with a laser light source at a wavelength of 515 nm, confocal microscopy was performed using different wavelength filters, as shown in Figure 3. Fluorescent images were obtained while using filters for 600 ± 37 nm and 530 ± 43 nm. Among them, intense red fluorescence was observed with 600 ± 37 nm laser light source (Figure 3b), indicating the fluorescence of FeAu NPs, which could be used to determine the fluorescent images of subsequent biological samples.

### 3.4. Analysis of Magnetic Properties of FeAu NPs and MMP-1 Antibody-Conjugated FeAu NPs

**Figure 4:**
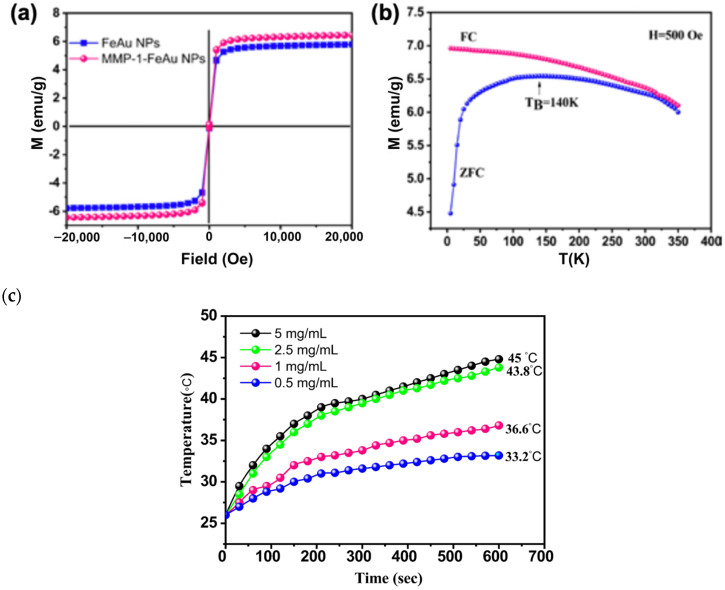
Magnetic properties of FeAu NPs as analyzed using SQUID. (**a**) ZFC/FC curve of FeAu NPs and MMP1-FeAu NPs (M-T curve), (**b**) hysteresis curves of FeAu NPs and MMP1-FeAu NPs (M–H curve) and (**c**) temperature elevation of the solution with the addition of different concentrations of FeAu NPs and stimulation with AMF over 10 min.

The magnetic properties of nanoparticles were evaluated using SQUID. The ZFC/FC curve showed the maximum at 140 K, which was the block transition temperature (TB). At temperatures lower than TB, the magnetization direction of the NPs is not consistent, and the amount of magnetization decreases with the temperature. The ferromagnetic substances loses its spontaneous magnetic moment and changes from an ordered ferromagnetic phase to a disordered paramagnetic phase (Figure 4a). The hysteresis curve passes through the origin, and there is no coercive force or residual magnetism, indicating that both FeAu NPs and MMP-1 antibody-conjugated FeAu NPs exhibit superparamagnetism; the saturation magnetization values of FeAu NPs and MMP1-FeAu NPs were found to be 5.8 and 6.5 emu/g, respectively, indicating that MMP-1 antibody conjugation had little effect on the saturation magnetization (Figure 4b).

### 3.5. Heat Generation upon Magnetic Field Stimulation

The main reason for using FeAu NPs in this study is their ability to generate hyperthermia upon magnetic stimulation. Importantly, cancer cells are more sensitive to heat than healthy cells. Furthermore, the cancer cells exhibit abnormal behavior or cellular death at temperatures ranging between 39–43 °C whereas healthy cells can survive temperatures as high as 46 °C [37]. For this experiment, FeAu NPs with different concentrations (0.5, 1, 2.5, and 5 mg/mL) were diluted in 1.5 mL DI water and exposed to AMF. The results revealed the relationship between the concentration of the NPs and the temperature elevation. At the concentration of 2.5 mg/mL, the solution temperature increased to 43.8 °C, elucidating the fact that a small concentration of the NPs is enough to and required to target cancer cells (Figure 4c). Furthermore, the temperature elevated to 45 °C at 5 mg/mL concentration of NPs, indicating that FeAu NPs are good carriers for magnetic heat therapy of cancer.

### 3.6. In Vitro Cytotoxicity Analysis

**Figure 5:**
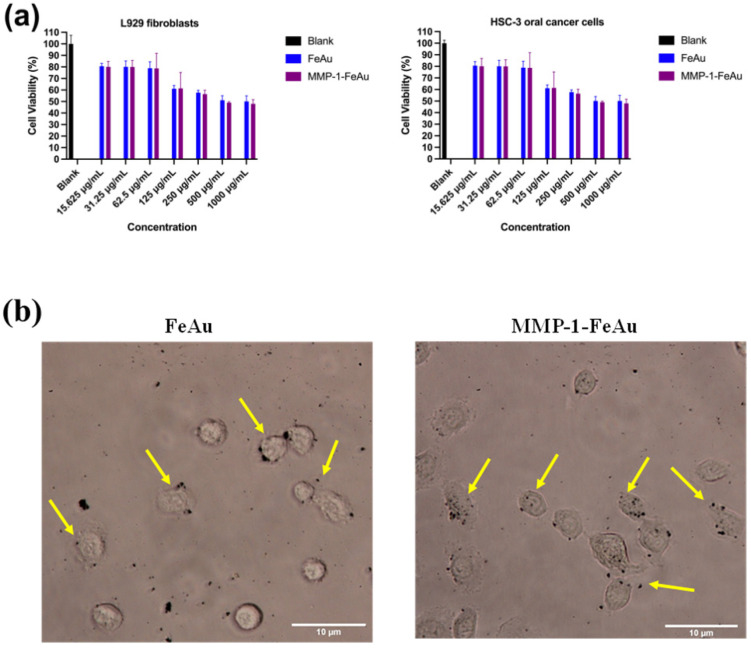

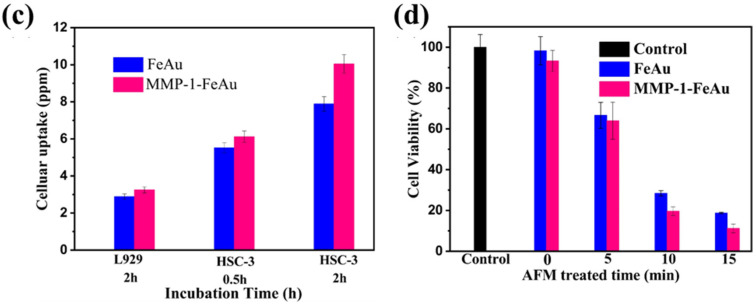
Cell responses of FeAu NPs and MMP-1 antibody-conjugated FeAu NPs. (**a**) Cellular viability of L929 and HSC-3 by the MTT assay (n = 3); (**b**) bio-TEM micrographs of human oral squamous carcinoma incubated with FeAu and MMP1-FeAu over different time periods; (**c**) quantification of cellular uptake of FeAu NPs and MMP1-FeAu NPs in HSC-3 cells and L929 cells analyzed by ICP-AES, (**d**) FeAu NPs and MMP1-FeAu NPs stimulated magnetically.

MTT assay was performed to evaluate in vitro cytotoxicity, which showed that both FeAu and MMP-1 antibody-conjugated FeAu NPs exhibited maximum bio-compatibility with a cell viability of 50% even at a concentration of 1 mg/mL (Figure 5a). No significant difference in the cell viability of L929 fibroblast cells was observed with Fe–Au and MMP1 antibody-conjugated FeAu NPs, indicating that MMP1 conjugation did not elevate cytotoxicity of the nanoparticles. Thus, MMP-1-FeAu NPs were considered suitable for further experiments. The present study aims to target HSC-3 cells using MMP1 antibody-conjugated FeAu NPs. Hence, the cell viability was evaluated by incubating HSC-3 cells in the presence of FeAu/MMP1-FeAu NPs and it was observed that the cell viability was inversely proportional to the concentration of Fe–FeAu/MMP1-FeAu NPs (Figure 5a). Moreover, the cell viability was decreased to almost 50% with MMP1-FeAu NPs at a concentration of 250 µg/mL. Notably, this concentration is one-fourth of the concentration used to evaluate L929 cell viability (1 mg/mL), demonstrating that the MMP1-FeAu NPs exhibit significant and specific cytotoxicity against HSC-3 carcinoma cell.

### 3.7. Analysis of Nanoparticle Ingestion Using Bio-TEM

We anticipated that the conjugation of MMP-1 antibody to FeAu NPs would enhance the cellular uptake of MMP-1-FeAu NPs, resulting in increased cell death. Therefore, time-based Bio-TEM was performed to verify the qualitative visualization of FeAu NPs and MMP-1-FeAu NPs endocytosis. As shown in Figure 5b, the Bio-TEM results showed the visibly enhanced ingestion of MMP-1 antibody conjugated FeAu NPs by HSC-3 cells as compared to FeAu NPs.

### 3.8. The Effects of NP-Mediated Hyperthermia on HSC-3 Cells

The uptake of FeAu NPs and MMP-1 antibody-conjugated FeAu NPs by HSC-3 and L929 fibroblast cell lines was analyzed using ICP-AES (Figure 5c). The result revealed that cellular uptake of MMP-1 antibody-conjugated NPs was higher (1.27-fold) than that of FeAu NPs. To investigate the effect of FeAu NPs-generated hyperthermia on the HSC-3 cell death, we incubated the cells with FeAu NPs and MMP-1 antibody-conjugated FeAu NPs, and stimulated both experimental groups with an external magnetic field. The time-based stimulation method was followed to determine the ideal time for which magnetic field exposure is needed to attain cancer cell death. As can be seen in Figure 5d, the cell survival rates of the FeAu NPs and MMP1-FeAu NPs groups decreased with increased magnetothermal treatment time. After 15 min of magnetic stimulation, the percentages of viable cells in FeAu NPs and MMP-1 antibody-conjugated FeAu NPs groups were 18% and 11%, respectively.

### 3.9. The Effects of NP-Mediated Magnetic Hyperthermia Therapy on Nude Mice

To study the therapeutic effects of NP-mediated magnetic hyperthermia on cancer cells, an in vivo mouse model of cancer was used, as described above. The human squamous-cell carcinoma (SCC) cells were injected subcutaneously into the hind limbs.

When the tumor volume reached around 40 mm^3^, the mice were randomly divided into three groups (N = 5 for each group) and intratumorally injected with FeAu NPs (0.2 mg/200 µL), antiMMP1-FeAu NPs (0.2 mg/200 µL) suspended in PBS, and PBS alone (control group). Further, after phagocytosis for 2 h, phagocytosis is the process where a cell engulfs the particle. Hereafter, to inhibit the tumor growth via the NPs injected, we used an alternating magnetic field (AMF, 700–1100 kHz) for 10 min. The average tumor volume was measured and recorded every third day for a period of 30 days after magnetic hyperthermia treatment.

**Figure 6:**
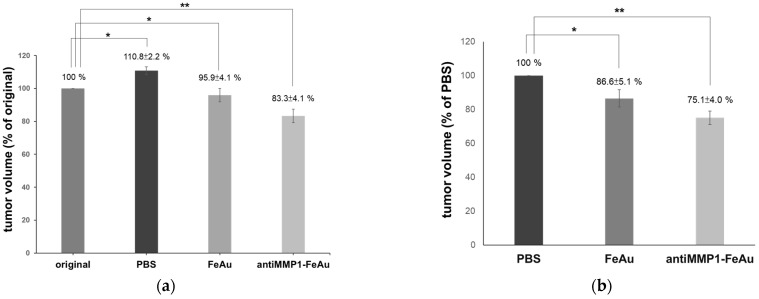
The in vivo anti-cancer effects magnetic heat after 30 days. (**a**) The tumor volume % of original; (**b**) the tumor volume % of PBS (control groups). Statistical analysis was performed using two-tailed student’s *t*-test and the level of significance was set at 0.05. * represents *p*-value ≤ 0.05 and ** represents *p*-value ≤ 0.01.

As shown in Figure 6 and Table 3, after 30 days of magnetic hyperthermia treatment, the tumor volume was increased by 10.9% in the experimental group treated with PBS only. In contrast, the tumor volume was reduced by 4.1% in the mice injected with FeAu NPs. The tumor volume of the antiMMP1-FeAu NPs group was reduced by 16.8% which highlights higher therapeutic efficacy of MMP-1-conjugated nanoparticles.

Based on the experimental results, it can be concluded that while the magnetothermal effect of FeAu NPs is sufficient for treating oral squamous tumors, FeAu NPs modified with antiMMP1 have superior therapeutic effects.

## 4. Conclusions

In the present study, we have developed MMP-1 antibody-conjugated iron–gold bimetallic NPs as a potential platform for hyperthermia-mediated cancer cellular death. The FeAu NPs were proved to be superparamagnetic in nature and capable of generating heat in a dose-dependent manner when exposed to AMF. Upon magnetic stimulation, MMP-1-FeAu NPs conjugate triggered cellular death in 89% of HSC-3 cells, confirming the efficacy of antibody-conjugated nanoparticles in limiting SCC growth. Further experiments confirmed a higher uptake of MMP-1 antibody-conjugated FeAu NPs by HSC-3 cells as compared to the L929 cells, confirming the efficacy of MMP-1-FeAu NPs in limiting SCC growth. Moreover, as the conjugate material displays autofluorescence and endocytosis, it might be used to diagnose cancers with a high concentration of MMP-1 in the future.

## Figures and Tables

**Table 1:** Elemental distribution analysis using EDS.

Element	Weight%	Atomic%
Fe	18.40	44.29
Au	81.60	55.71

**Table 2:** Molar ratio analysis using ICP.

Element	Atomic%	Molar Ratio%
Fe	49	0.96
Au	51	1.04

**Table 3:** Tumor volume change in animal study by magnetic hyperthermia therapy.

	Volume (mm^3^)	Variation (%)
Original (before magnetic heat treat)	39.5 ± 0.6	-
Control (magnetic heat treat after 30 day)	43.79 ± 1.5	+10.9
FeAu NPs (magnetic heat treat after 30 day)	37.87 ± 1.0	−4.1
antiMMP1-FeAu NPs (magnetic heat treat after 30 day)	32.88 ± 1.3	−16.8
